# Pyogenic liver abscess following perforated appendicitis

**DOI:** 10.1590/0037-8682-0244-2022

**Published:** 2022-09-30

**Authors:** Behiç Akyüz

**Affiliations:** 1 Bursa City Hospital, Department of Radiology, Bursa, Turkey.

**FIGURE 1:**
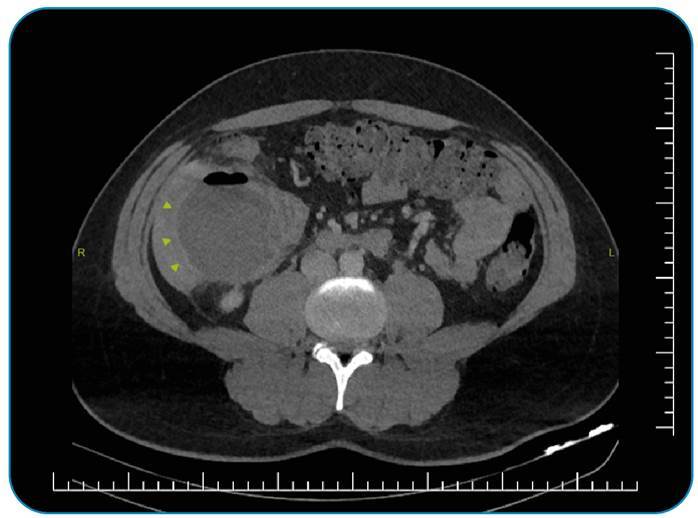
CT shows right hepatic lobe subcapsular abscess (arrowheads).

**FIGURE 2:**
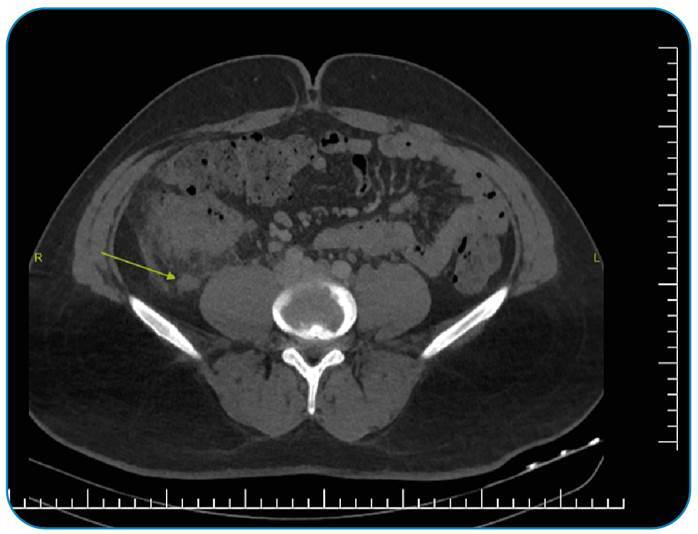
Acute appendicitis (arrow) can be observed.

A 26-year-old male patient presented to the emergency department with abdominal pain, fever, weakness, and loss of appetite, which he had been experiencing for approximately four days. Laboratory studies revealed leukocytosis (19 × 103 µL) and an elevated C-reactive protein level (328 mg/L). Computed tomography (CT) showed a subcapsular abscess in the right hepatic lobe (Figure 1). We observed that the appendix was attached to the liver capsule and opened to the liver with a fistula (Figure 2). Ultrasound-guided percutaneous drainage of the subcapsular liver abscess was performed. Following antibiotic treatment, the patient was discharged for outpatient follow-up and considered for an appendectomy.

Pyogenic liver abscess is extremely rare. It can lead to death if left untreated^1^. Following the diagnosis, appropriate treatment should be applied with great care, pylephlebitis should be kept in mind, and portal vein involvement should be considered in imaging^2^.
